# Genomic-Based Early Detection Screening: A Literature Review of Prospective Trials and Emerging Strategies for Gastrointestinal Cancers

**DOI:** 10.7759/cureus.68881

**Published:** 2024-09-07

**Authors:** Eemon Tizpa, Kaveh Sharzehi, Nima Nabavizadeh

**Affiliations:** 1 Radiation Oncology, Washington State University Elson S. Floyd College of Medicine, Spokane, USA; 2 Gastroenterology and Hepatology, Oregon Health and Science University School of Medicine, Portland, USA; 3 Radiation Oncology, Oregon Health and Science University School of Medicine, Portland, USA

**Keywords:** circulating tumor dna (ctdna), multicancer detection, genomic cancer screening, multicancer early detection test, gastrointestinal cancer, cancer screening

## Abstract

Numerous genomic-based early detection screening tests are being developed. These tests have the potential to revolutionize current single-organ screening paradigms, especially in gastrointestinal cancers. In this review, we underscore the performance of these genomic-based early detection tests based on prospective clinical trials. Moreover, we discuss a professional advancement for gastroenterologists in the diagnostic assessment of individuals who are cancer signal positive.

## Introduction and background

Cancer continues to be a leading cause of death worldwide. Over the next 20 years, the incidence of cancer is projected to increase by nearly 50%, resulting in approximately 28 million new cases annually [1]. This rise is attributed to population growth, longer patient lifespans, obesity, and heightened environmental exposures due to the worsening effects of climate change. Moreover, many cancers are diagnosed when the patient is symptomatic or the disease has spread, necessitating more aggressive treatments and resulting in poorer prognosis and quality of life for the patient [2]. Presently, there are only four cancer screening protocols with level A or B recommendations by the USPSTF (breast, lung, cervical, and colorectal cancers (CRCs)). These protocols focus on specific cancers and exhibit varying degrees of adoption and compliance. These protocols target individual cancers and have varying levels of adoption and compliance [3]. Also, some screening tests, like those for breast and cervical cancer, have a low positive predictive value (PPV) and often yield frequent false-positive results [4].

One cancer that has shown improved outcomes with earlier detection is CRC. CRC stands as the third most prevalent cancer globally, accounting for 10% of all cancer cases, and ranks as the second leading cause of cancer-related deaths worldwide [5]. Early detection of CRC has a significant impact on overall patient survival: the five-year survival rate is 91% for individuals with localized disease, compared to only 14% for those with metastatic disease [6]. Asymptomatic screening methods have been proven to significantly reduce deaths from CRC. However, adherence to these methods remains notably low. Only about 59% of eligible patients aged 45 and older follow the colorectal screening guidelines, falling short of the national target of 80% [6,7]. Factors contributing to this include cognitive-emotional factors such as lack of education about CRC, fear of procedure complications, and modesty of the procedure, as well as barriers to healthcare access and a history of chronic disease (i.e., diabetes, hypertension) [8,9].

Because of these shortcomings with current CRC screening paradigms, there is a demand for noninvasive cancer screening modalities to address these trends. Currently, available stool-based and blood-based CRC screening tests are cost-effective and noninvasive, yet their PPV and false positive rates vary significantly, impacting the quality of patient care and limiting their overall effectiveness [10]. The use of a genomic-based cancer screening test capable of simultaneously detecting and localizing multiple cancer types could help address this unmet need. Multi-cancer early detection (MCED) tests are currently being investigated and are in the early phases of development but are showing promise in early cancer detection. For MCEDs to create an impact on cancer deaths, they will need to have high sensitivity to detect clinically relevant malignancies early and avoid indolent malignancies but also high specificity to avoid unnecessary invasive follow-up procedures/tests for the patient. This review will examine the latest data on various genomic-based early detection tests (e.g., MCED), highlighting their potential benefits as supplementary screening methods to enhance cancer detection rates. Additionally, we propose a professional expansion for gastroenterologists concerning the diagnostic evaluation of individuals who test positive for cancer signals.

Methods

This is a literature review. The databases PubMed, Google Scholar, ClinicalTrials.gov, and Science Direct were searched through the keywords “Multicancer Early Detection,” “Early Cancer Detection,” “Gastrointestinal cancer screening,” “Cancer screening,” “ctDNA screening,” “FIT/FOBT,” and “Genomic Cancer screening”; study dates range from 2014 to 2024.

## Review

Multi-cancer early detection tests

MCEDs have the capability to detect multiple types of cancers (including gastrointestinal cancers) from a single sample from blood, urine, stool, or other sources. These tests work to analyze the sample for specific DNA, proteins, or other biomarkers released into the circulation by cancer cells. If these markers are identified, it may indicate the presence of cancer and potentially reveal the tissue of origin (TOO) [11]. Most of these genomic tests are currently being investigated for their safety and efficacy in the early stages of development. While numerous early MCED studies exist, we will focus on examining two companies, Exact Science and GRAIL, that have progressed the furthest along with prospective studies in MCED devices, while also analyzing organ-specific genomic assays for gastrointestinal cancers.

CancerSeek

Exact Science has developed CancerSEEK, an MCED that identifies circulating tumor DNA (ctDNA) and protein biomarkers that can identify eight common cancer types (breast, esophagus, colon/rectum, liver, lung, ovary, pancreas, and stomach). In a retrospective study of 1,005 patients with the prior listed cancer types, Cohen et al. showed that CancerSEEK displayed a sensitivity between 33% and 98% across the eight cancer types, with increased sensitivity for later cancer staging (stage I cancers: 40%; stage III cancers: 80%). With this sensitivity, they demonstrated an overall specificity of >99% [12].

After further development of the CancerSEEK test, a prospective trial, DETECT-A, studied the outcome of CancerSEEK while clinically validating it with PET/CT in nearly 10,000 women aged 65-75 with no prior cancer history [13]. Patients had their blood drawn and analyzed via the CancerSEEK test; positive blood tests would be confirmed with PET/CT testing to validate the test and determine TOO. Among the 9,911 patients tested, 5% (n = 490) had a positive blood test, and 1.4% (n = 134) were confirmed positive with PET/CT. Within the confirmed test cohort, 50% (n = 65) of patients had radiographic imaging indicative of cancer. Among these, 41% (n = 26) underwent biopsies, resulting in 25 cancer diagnoses. Additionally, 24 more cancers were diagnosed through standard-of-care screening methods, with half of these cases showing positive baseline blood tests. For CancerSEEK, its sensitivity and specificity were 27% and 99%, respectively, with a PPV of 41%.

A follow-up retrospective case-control study used CancerSEEK to assess for cancer DNA mutations, methylation, aneuploidy, and protein abnormalities in nearly 2,400 samples [14]. Combining all these biomarkers, 2,386 samples were analyzed, yielding an overall sensitivity of 53% and specificity of 98.7%. Similarly to Cohen et al. findings, sensitivity increased with later stages of cancer (I/II: 32.8%, III: 60%, IV: 87.7%). Further validation of this study is being completed in the ongoing ASCEND trial (NCT04213326). In 2024, Exact Sciences rebranded the CancerSEEK test as Cancerguard.

Galleri

The Galleri test by GRAIL has shown significant promise. In a prospective, case-control sub-study (NCT02889978 and NCT03085888), Galleri was validated with Circulating Cell-Free Genome Atlas (CCGA), specifically based on circulating cell-free DNA (cfDNA) methylation profiles [3]. Specimens were collected from patients with newly diagnosed cancer prior to therapeutic intervention, as well as from healthy controls. The initial sub-study identified methylation as the primary focus for further assay development. With this updated targeted methylation assay for cfDNA fragments, Liu et al. began the second CCGA sub-study by dividing up 6,689 samples (patients with and without cancer) into training and validation sets to determine test validation and TOO accuracy [3]. The validation set displayed a specificity of 99.3% with a false positive rate (FPR) of <1%. Sensitivity was 76% for 12 high-signal cancers (anus, bladder, colon/rectum, esophagus, head and neck, liver/bile duct, lung, lymphoma, ovary, pancreas, plasma cell, and stomach) while overall sensitivity was 55%. Signal TOO was identified in 96% of samples with a 93% accuracy. In the final validation study of the CCGA sub-study, Klein et al. detected cancers across more than 50 types with a specificity of 99.5% and accurately predicted TOO in 1273 (89%) of 1435 participants [15]. Overall sensitivity for cancer detection was 52%, varying by cancer type and stage. PPV was found to be 44%, which is significantly higher than most standard-of-care cancer screening tests (e.g., FIT and mammography) [16,17].

Based on these CCGA validation results, PATHFINDER-1 (NCT04241796), a prospective return-of-results cohort study, evaluated 6,621 healthy, asymptomatic patients over 50 years of age [18]. The results revealed a cancer detection rate of 1.4% (n = 92), resulting in 36 cancer diagnoses in 35 patients, of which 26 cancers were lacking standard-of-care screening [18]. PPV was recorded at 38% with an NPV of 99%, with a specificity of 99.1% and an 87% accuracy for predicting TOO. Notably, the MCED assay detected various cancer types for which no screening exists, including some found at early stages. For instance, early-stage cancers of the bile duct, small intestine, and pancreas, which are amendable to surgical resection, were detected.

Following PATHFINDER-1, the SIMPLIFY (ISRCTN10226380) trial was a prospective observation study in England that used ctDNA analysis with Galleri MCED in symptomatic patients referred by their primary care physician (PCP) [19]. Approximately 5,460 participants were analyzed, with the Galleri MCED test detecting cancer signals in 323 cases, of which 244 cases were subsequently confirmed as cancer diagnoses. Sensitivity and specificity were found to be 66% and 98%, respectively, with a PPV of 76%, NPV of 98%, and predicted TOO at 85% accuracy [19]. Interestingly, in upper gastrointestinal cancers, the sensitivity and NPV were 80% and 99%, respectively. This infers that MCEDs such as Galleri could be specifically advantageous for detecting gastrointestinal cancers.

Currently, UK-NHS is conducting a randomized prospective Galleri study (ISRCTN91431511) to evaluate test performance in 140,000 adults assigned to either standard-of-care screening only or standard-of-care supplemented with annual MCED testing (Table 1). The trial has completed its enrollment phase. The primary goal is to reduce the incidence rate of stage III and IV cancers diagnosed in the intervention arm compared to the control arm within 3-4 years following randomization [20].

Colon Cancer-Specific Early Detection Tests

A multitude of CRC screening modalities exist. Guaiac-based tests utilize hydrogen peroxide to turn the color of the stool sample when in contact with blood. Many trials of guaiac-based tests have demonstrated that early detection of CRC can lead to successful treatment and a reduction in mortality from the disease [21]. With these benefits, a follow-up study by Carethers described how these tests had poor sensitivity, as low as 50%, and did require dietary alterations prior to testing [22]. Another stool test, fecal immunochemical testing (FIT), has been identified to have better sensitivity and specificity compared to guaiac-based tests and does not require dietary interventions [22]. Similar to guaiac-based tests, however, FIT has been recommended to be performed annually per USPSTF.

In 2014, progress in noninvasive multitarget stool DNA testing began, incorporating both DNA molecular markers and hemoglobin level assessment. One such test is Exact Sciences’ Cologuard, which received FDA approval and was included in CRC screening guidelines for average-risk patients [23]. In the DeeP-C trial (NCT01397747), Cologuard was shown to be superior to FIT in the detection of CRCs (sensitivity of 92% vs. 74%) and advanced precancerous lesions (sensitivity of 42.4% vs. 23.8%), yet its specificity was much lower than FIT alone (87% vs. 95%) [24].

Two studies published in the *New England Journal of Medicine *in March 2024 demonstrated additional potential in enhancing the utilization of CRC screening. The BLUE-C study (NCT04144738) investigated the performance of Cologuard and compared this with FIT in more than 20,000 participants who were at average risk, undergoing standard-of-care screening [25]. Out of all the participants, only 98 (0.5%) cases of CRC were identified. Cologuard demonstrated higher sensitivity compared to FIT in detecting CRC (94% vs. 67%), advanced precancerous lesions (43% vs. 23%), and high-grade dysplasia (75% vs. 47%) [25]. While Cologuard showed an improved specificity of 91% for advanced neoplasia, it still remains lower than FIT, which had a specificity of 95%. The higher specificity should increase the detection efficiency by reducing the false positive rate and lowering the number of follow-up colonoscopies.

The ECLIPSE study (NCT04136002) investigated the testing performance of a cfDNA blood-based study, Shield manufactured by Guardant Health, for CRC in an average-risk population [26]. Plasma cfDNA was obtained from 7861 participants (45 to 84 years old) with a sensitivity of 83% in detecting CRC, a specificity of 90% for advanced neoplasia, and a sensitivity of 13.2% for advanced adenomas. Although the sensitivities observed in this study are lower than those of DNA stool assays, the specificities are comparable. Additionally, because these assays are blood-based, they could be more easily incorporated into routine blood draws at clinics, thereby potentially increasing the prevalence of CRC screening across various populations.

Freenome Holdings, another major biotechnology company, recently released results from their blood-based CRC test conducted through the PREEMPT CRC trial (NCT04369053). This prospective registrational study aimed to validate Freenome’s blood-based assay for the early detection of CRC in average-risk adults. With nearly 27,010 participants, the Freenome blood test demonstrated 79% sensitivity in detecting CRC (stage I: 57%; stage II: 100%; stage III: 82.4%; stage IV: 100%), 12.5% sensitivity in detecting advanced adenomas, and a specificity of 92% for non-advanced colorectal neoplasia [27]. This trial worked on using a hybrid model that incorporates both virtual and traditional recruitment methods to include underserved communities and develop a more representative population. With a significantly larger participant size, Freenome’s assay exhibited relatively lower sensitivity and specificity compared to Shield. However, due to the broader diversity of its patient population, Freenome’s assay is more representative of the overall patient demographic.

Other Gastrointestinal Early-Detection Tests

Alongside colorectal detection, the K-DETEK study (NCT05227261) enrolled 10,000 patients to evaluate the efficacy of SPOT-MAS in detecting five common cancers, including CRC and gastric cancer in Vietnam [28]. The study found that the ctDNA test detected these cancers in asymptomatic individuals with a PPV of 60% and a TOO accuracy of 83.3%. This study demonstrated the clinical applicability of this MCED test in a low-to-middle-income country like Vietnam, providing a more representative patient population for test validation.

In addition, Helio Genomics sponsored the prospective study CLiMB (NCT03694600), which enrolled 1,600 participants to validate the performance of HelioLiver Dx in detecting hepatocellular carcinoma (HCC) in patients with liver cirrhosis [29]. Preliminary results indicate that the HelioLiver Dx test has shown higher sensitivity than ultrasound in identifying early-stage HCC lesions. The final results will be available upon the trial's completion in July 2024.

Ongoing genomic-based prospective trials are listed in Table 1. Notably, the upcoming ACCESS trial, sponsored by the National Cancer Institute, will carry out a multi-center randomized controlled trial to evaluate the feasibility of using MCEDs as a screening modality [30].

**Table 1:** Ongoing trials for genomic-based early detection tests MCED: multicancer early detection

Clinical trial ID	Sponsor	Trial name	Estimated enrollment	Condition	Age eligibility (years)	Status	Estimated date of completion
NCT05366881	Adela Inc	CAMPERR	7,000	MCED	≥40	Recruiting	2026-12
NCT05227534	Burning Rock	PREVENT	12,500	MCED	40-75	Recruiting	2028-12-31
NCT04825834	DELFI Diagnostics	DELFI-L101	2,660	Lung	≥50	Recruiting	2026-05-31
NCT05306288	DELFI Diagnostics	CASCADE-LUNG	15,000	Lung	≥50	Active, not recruiting	2025-03-31
NCT06122077	Freenome	PROACT-LUNG	20,000	Lung	≥50	Recruiting	2027-06-15
NCT05516927	Freenome	Sanderson Study	8,000	MCED	≥30	Recruiting	2025-09-06
NCT05254834	Freenome	Vallania Study	5,400	MCED	≥30	Recruiting	2025-06-30
NCT05155605	GRAIL	PATHFINDER-2	35,000	MCED	≥50	Recruiting	2027-12-31
ISRCTN91431511	GRAIL	Galleri-UK NHS	140,000	MCED	50-77	Active, not recruiting	2026-02-28
NCT05205967	GRAIL	REFLECTION	17,000	MCED	≥22	Recruiting	2026-08-23
NCT03085888	GRAIL	STRIVE	99,481	Breast	≥18	Active, not recruiting	2025-05
NCT03934866	GRAIL	SUMMIT	13,035	Lung	55-77	Active, not recruiting	2030-08
NCT03774758	Guardant Health	17–22915	590	Lung	≥40	Recruiting	2024-12-31
NCT05181826	Helio Health	ELITE	1,200	Cancer-free, benign	≥18	Recruiting	2025-12
NCT05199259	Helio Health	LIVER-1	1,200	Liver	≥18	Recruiting	2025-03
NCT05432128	Singlera Genomics	2019YFC1315803	600	Lung	18-75	Recruiting	2025-12-31
NCT05485077	Singlera Genomics	KYS-2022007	18,000	Colorectal	≥40	Recruiting	2028-07-09

Genomic assay limitations

To achieve widespread adoption of MCED testing, several challenges must be addressed. These blood tests are expected to be conducted in primary care and community settings to enhance access and equity for patients from medically underserved populations. However, this necessitates that medical practitioners prescribing these MCED tests receive comprehensive training. They must fully understand cancer screening principles and MCED performance, be able to clearly explain MCED test results, and effectively facilitate diagnostic evaluations for patients who require further examination.

These genomic assays have demonstrated a high specificity of approximately 98%, but their sensitivities have been as low as 33%. While the sensitivities of these tests improve with advanced cancer stages, there is a concern that early-stage cancers, which would benefit most from treatment, might be overlooked [31]. These tests could impose a similar burden to that posed by standard-of-care screening method, which can lead patients to question the benefit of these blood-based assays. For Galleri and CancerSEEK, the false positive rates were <1%, compared to the USPSTF standard-of-care screening, which has rates between 10% and 15% [5,16].

Many clinical trials, such as DETECT-A and PATHFINDER-1, had short follow-up periods of only 12 months, which may be insufficient for detecting most cancers, potentially affecting the current false positive results for these genomic detection tests. The ongoing PATHFINDER-2 study aims to address this issue by following patients for three years, providing more comprehensive longitudinal data. Another factor to consider is the lack of diversity in some of these earlier trials. The patient population in PATHFINDER-1 was 90% white, suggesting that the benefits of Galleri’s test may not be representative of underrepresented populations [18]. To address this, PATHFINDER-2 has incorporated strong diversity endpoints in its protocol.

The cost of these genomic-based tests will be another limiting factor in their availability to patients. Currently, these genomic detection assays have not been FDA-approved or authorized by the Clinical Laboratory Improvement Amendments of 1988 (CLIA). While some genomic-based tests have been released as laboratory-developed tests (LDTs) or have received Breakthrough Device Designation from the FDA, they still have not secured reimbursement from the Centers for Medicare & Medicaid Services [32]. Consequently, health insurance does not cover these tests at present. Given this, the cost of these tests can be approximately $900. Such high costs can further reduce adherence and exacerbate existing healthcare inequities.

Another limitation to consider is whether easier access to these genomic detection tests will lead to decreased adherence to current standard-of-care screening modalities. It is theorized that patients might opt for less invasive MCED testing, such as a blood draw, over more invasive standard screening procedures, potentially leading to reduced adherence to standard screening recommendations. Fortunately, current prospective MCED studies do not indicate this concern. In a study of 10,000 women screened by MCED, adherence to mammography remained at 99%. Additionally, another MCED study reported minimal impact on planned screening after patients received their test results [13,18].

Interestingly, although the cancer incidence rate has increased, likely due to more screening modalities, the mortality rate has decreased by 2.1% [5]. The introduction of more and newer screening methods brings concerns about over-detection and overtreatment of indolent cancer types. One article reported that approximately 25% of breast cancers, 55% of prostate cancers, and 55% of melanomas have been overdiagnosed, leading to unnecessary physical and psychological harm [33]. Fortunately, since these MCED tests primarily detect the shedding of tumor fragments (cfDNA or ctDNA), they may be more effective at identifying lethal cancers rather than indolent ones that do not impact overall patient survival. A large MCED case-control study found that detected cancers had survival outcomes comparable to those observed in Surveillance Epidemiology and End Results (SEER) data, while cancers that were not detected had better than expected outcomes for all stages [34]. With further confirmation, these test features would minimize the risks of over-diagnosis.

Genomic assays in gastrointestinal cancers: opportunities for gastroenterologists

Gastrointestinal cancers make up around 25% of cancer incidence and 33% of cancer-related deaths globally [35]. New machine-learning models have shown high accuracy in detecting early-stage upper gastrointestinal cancers with endoscopic images [36]. Previous studies have shown that early cancer diagnosis at stage I/II, before local and regional spread, is associated with improved survival and reduced mortality, as treatments are better tolerated [5,37]. Currently, there are no recommended screening guidelines for the general population or low-risk individuals for upper gastrointestinal cancers such as those of the esophagus, stomach, liver, and pancreas. Screening for these cancers typically occurs in response to symptoms, which are often vague, making the diagnostic process quite challenging.

Colonoscopies have a low adherence rate. Stool-based test screenings have shown promise in increasing adherence to CRC monitoring. However, adherence to repeated stool-based testing declines over time. A 2019 systematic review found that adherence to repeated fecal occult blood (FOBT) screening was 60.3% for two consecutive rounds of screening but dropped to 0.8% after three rounds [38]. A retrospective study of US health system data from 2007 to 2008 showed that initial adherence to FIT was 47% but dropped to 24% over four years of screening [39]. This data highlights the need for alternative testing modalities to increase patient adherence to CRC screening.

Genomic-based early detection tests such as MCEDs could provide an excellent adjunct screening method that is simple, non-invasive, and particularly beneficial for community clinics, especially in areas with lower adherence rates to CRC screening compared to the general population. In the final analysis of the CCGA sub-study, Klein et al. demonstrated that Galleri's TOO accuracy was 82% [15]. With a specificity of 99.5%, the MCED test can detect both colorectal and rectal cancers at earlier stages, before metastasis. In a randomized controlled trial (NCT03598166) comparing the reoffering of colonoscopies/FIT alone versus offering a genomic blood test to individuals who declined colonoscopies/FIT, investigators found that the genomic blood test increased screening by 7.5% among patients who declined colonoscopies/FIT without reducing the uptake of first-line screening options [40].

The use of genomic-based early detection tests could be highly beneficial in a gastroenterologist's practice to detect these insidious gastrointestinal cancers early on, especially in signal-positive patients. With a PPV of 40-80% in the Galleri test, performing further diagnostic modalities by gastroenterologists could be considered to catch gastrointestinal cancers sooner. In the SIMPLIFY trial, the Galleri test performed highest in gastrointestinal cancers, especially esophageal cancer, with a sensitivity of 96% [19]. The organ-specific blood-based tests in the ECLIPSE and PREEMPT CRC trials showed a sensitivity ranging from 79% to 83%, while the K-DETECT study showed the blood-based assay had a PPV of 60% in gastric and CRC patients.

If genomic-based early detection tests are proven to have clinical additive benefits with standard-of-care screening modalities, a potential clinical scenario could involve a PCP performing this test on patients with vague symptoms (e.g., weight loss, dysphagia) during routine blood draws. The PCP would then relay signal-negative findings to over 98% of patients, with 1-2% of patients with signal-positive findings to see a gastroenterologist to perform further diagnostic workup. Once the cancer is diagnosed, gastroenterologists can refer to oncology to determine patient's treatment plan.

## Conclusions

In conclusion, genomic-based early detection assays have the potential to overcome current limitations in cancer screening. Numerous prospective studies are underway to validate and demonstrate the benefits of these tests in reducing cancer morbidity. Gastroenterologists can play a crucial role in identifying patients with vague gastrointestinal symptoms and guiding them through further diagnostic workups and eventual treatments. By utilizing genomic-based early detection assays, specialized providers like gastroenterologists can support PCPs, thereby minimizing delays from diagnosis to treatment initiation.
